# Modulation of Instagram Number of Followings by Avoidance in Close Relationships in Young Adults under a Gene x Environment Perspective

**DOI:** 10.3390/ijerph18147547

**Published:** 2021-07-15

**Authors:** Andrea Bonassi, Alessandro Carollo, Ilaria Cataldo, Giulio Gabrieli, Moses Tandiono, Jia Nee Foo, Bruno Lepri, Gianluca Esposito

**Affiliations:** 1 Department of Psychology and Cognitive Science, University of Trento, 38068 Rovereto TN, Italy; andrea.bonassi@unitn.it (A.B.); alessandro.carollo@studenti.unitn.it (A.C.); ilaria.cataldo@unitn.it (I.C.); 2 Mobile and Social Computing Lab, Fondazione Bruno Kessler, 38123 Trento TN, Italy; lepri@fbk.eu; 3 Psychology Program, School of Social Sciences, Nanyang Technological University, Singapore 639818, Singapore; giulio001@e.ntu.edu.sg; 4 Lee Kong Chian School of Medicine, Nanyang Technological University, Singapore 308232, Singapore; mosestandiono@ntu.edu.sg (M.T.); jianee.foo@ntu.edu.sg (J.N.F.); 5 Human Genetics, Genome Institute of Singapore, Singapore 138672, Singapore

**Keywords:** gene x environment, close relationship, avoidance, serotonin transporter gene, rs25531, social networking sites, online behavior, Instagram

## Abstract

Social networking sites have determined radical changes in human life, demanding investigations on online socialization mechanisms. The knowledge acquired on in-person sociability could guide researchers to consider both environmental and genetic features as candidates of online socialization. Here, we explored the impact of the quality of adult attachment and the genetic properties of the Serotonin Transporter Gene (*5-HTTLPR*) on Instagram social behavior. *Experiences in Close Relationships-Revised* questionnaire was adopted to assess 57 Instagram users’ attachment pattern in close relationships with partners. Genotypes from the *5-HTTLPR*/rs25531 region were extracted from the users’ buccal mucosa and analyzed. Users’ Instagram social behavior was examined from four indexes: number of posts, number of followed users (“followings”) and number of followers, and the Social Desirability Index calculated from the followers to followings ratio. Although no interaction between rs25531 and ECR-R dimensions was found, an association between avoidance in close relationships and Instagram number of followings emerged. Post hoc analyses revealed adult avoidance from the partner predicts the Instagram number of followings with good evidence. Moreover, users reporting high avoidance levels displayed fewer followings than users who reported low levels of avoidance. This research provides a window into the psychobiological understanding of online socialization on Instagram.

## 1. Introduction

Human social life has faced a massive revolution since the beginning of the 21st century [1,2]. The advent of social media and social networking sites has boosted novel opportunities to interact with people, realizing the so-called “culture of connectivity” [3,4]. Among the most popular social networking sites (SNSs), Instagram is a widely used platform that allows users to share multimedia contents (e.g., videos, photos and daily stories), together with a messaging service for users who wish to communicate with each other [5]. As a matter of fact, there are also multiple factors associated with its usage. For instance, Instagram usage can lead to positive conditions, such as lowering feelings of loneliness [6]. Another branch of research reports that Instagram use is linked to an increase in depressed mood [7,8,9], poorer sleep quantity and quality [10] and potentially lower body image satisfaction in conditions of social comparisons with peers [11,12,13].

However, the mechanisms underlying users’ social behaviors on platforms, such as Instagram, are still being discussed. In fact, it is not clear whether online socialization is influenced by the same determinants of in-person social interaction. Some studies documented the key-role of developmental, social, affective, and environmental factors on the modulation of Instagram social behaviors [14,15,16]. The same in-person adult attachment with peers or with the partner apparently drives users’ social attitudes on the SNS [17,18]. For instance, weak feelings of peer belonging encourage the adoption of deceptive-like seeking behaviors on the platform [16]. As a matter of fact, a relationship seems to exist between the attachment status measured by the Experience in Close Relationships questionnaire [19] and the behavioral patterns that lead to SNSs addiction [20].

Nevertheless, only a few studies have addressed the comprehension of the virtual social life by taking into account the genetic outlook [13]. Twin studies have proved that both genetic and unshared environmental influences may regulate social media behavior and atypical or problematic internet usage [21,22,23,24], but it is not clear what the specific genetic mechanisms underlying these processes are.

Several neurotransmitters are involved in the regulation of human social cognition and behavior [25,26,27]. In particular, serotonin seems to be crucial for social learning and interpersonal behavior, being positively associated with social dominance [28,29] and negatively correlated with aggression [30,31]. Altered levels of serotonin are responsible for social deficits, such as those evident in the core symptoms [32,33] of the Autism Spectrum Disorder [34,35].

Levels of serotonin in the brain are modulated by the Serotonin Transporter Gene (SLC6A4). In particular, the re-uptake of the molecules of serotonin from the synaptic cleft depends on the form of the promoter region of the Serotonin Transporter Gene (*5-HTTLPR*) [36,37,38]. Within the promoter region of the Serotonin Transporter Gene, the rs25531 Single Nucleotide Polymorphism (SNP) confers variability among people in regards to two allelic forms: the thymine (T) to cytosine (C) substitutions with the paired nucleotides adenine (A) to guanine (G). The debate around which allele within this SNP would confer a higher susceptibility to the social environment is still open. For instance, Schneider et al. [39] documented that individuals with a diagnosis of Major Depressive Disorder carrying at least one G allele on the *5-HTTLPR*/rs25531, when exposed to high stressful experiences, showed greater amygdala reactivity to threat and lower methylation when compared to A/A. Conversely, mental health of adult carrying T/T variation seems to be regulated by parents’ social support when compared to C-carriers [40]. Different studies also found that SLC6A4 polymorphisms may be related with personality traits (e.g., Openness to Experience) [41] and disorders, such as the antisocial personality disorder [42,43,44].

To date, two studies have already brought insights into the mechanisms underlying Instagram social behavior by considering the rs25531 genotype under a gene × environment approach. Specifically, Bonassi et al. [45] showed that participants with the pair T/T displayed a higher level of general sociability than C-carriers when exposed to positive maternal care in the early period of life. At the same time, Bonassi et al. [46] recorded a cross-interaction of genotype and confidence towards peers on the number of followed users, thus discovering that T/T individuals showed lesser followings than C-carriers. Overall, the rs25531 genotype is known to interact with the quality of early caregiving and adult relationships with peers in modulating Instagram social behavior.

### Aim and Hypothesis

To broaden the confined knowledge on the psychobiological properties of online socialization, this research frames Instagram in the context of gene × environment interactions. This framework allows us to overcome the classic polarization of nature and nurture, to obtain an integrated perspective of human development [47,48]. Given the direct genetic involvement of SLC6A4 expressions in social development [49] and antisocial behavior [50], we assume that rs25531 could be indirectly associated with online social processes. In agreement with former outcomes [45,46], we also assume that the attachment status could moderate the effect of rs25531 on the Instagram behavior. Therefore, the current work probes the potential implication of rs25531 and adult attachment on different Instagram social attitudes. As in a previous work [51], Instagram users were asked to report their levels of anxiety and avoidance towards a partner by answering the Experiences in Close Relationships-Revised questionnaire. Genetic data were extracted from users’ buccal mucosa cell samples and assessed with regards to the genetic region rs25531. Finally, three indexes were collected from users’ Instagram profiles: number of posts, followed people (“followings”) and followers. A further index was derived from the followers to followings ratio, called Social Desirability Index [52]. Three Instagram indexes—number of posts, followings and followers—were selected for the hypothesis based on previous findings [45,46,51,52], whereas Instagram number of followers was inspected at an exploratory level. The following hypothesis is proposed: Instagram users with the genetic factor more sensitive to life stressors (rs25531 T/T) would show a lower Instagram activity, in regards to the number of posts, followings and Social Desirability Index (SDI), when they experience a negative and untrustworthy relationship with the partner (low scores in the ECR-R dimensions Avoidance and Anxiety) compared to less vulnerable genetic carriers (rs25531 C-carriers). In line with previous works [51], gender was not expected to moderate such interaction.

## 2. Materials and Methods

### 2.1. Participants

A sample of 61 non-parent Singaporean students from the Nanyang Technological University (Singapore) was involved in the data collection procedure. All participants (*a*) were Instagram users, (*b*) aged between 18 and 30 years old, and (*c*) no current or past history of genetic, neurological or psychiatric disorders. Within this pool, 4 participants with missing or corrupted data were excluded. A total of 57 Instagram users (41 females with mean age = 20.24; 16 males with mean age = 22.56) were considered for the analysis [53].

### 2.2. Assessment

A three-step procedure was designed to gather different classes of data. At step one, the participants compiled an online questionnaire (e.g., the Experiences in Close Relationships-Revised) which assessed their attachment expectations with a partner in close relationships. At step two, participants’ buccal mucosa was collected by cotton swabs, and the genotype was analyzed in the laboratory to obtain the *5-HTTLPR*/rs25531 genetic profiles. At step three, an ad hoc Python scraper [54] was applied to extract three Instagram indexes starting from the Instagram profile of each user: number of published posts, number of followings, and number of followers. Neither images or information about individual published posts were obtained. In the event in which the scraper failed to obtain data for any reason (e.g., private profile, connection error, wrong username, etc.), data were manually corrected. A comparable assessment across the three steps was adopted in a previous study [51].

### 2.3. Attachment in Close Relationships

Instagram users’ adult attachment style with a romantic partner was assessed through the *Experiences in Close Relationships-Revised* (ECR-R) developed by Fraley et al. [55] (average Cronbach’s α=0.89). The ECR-R is a well-recognized self-report questionnaire, which counts 36 items with answers expressed on a Likert 7-point scale, and that has proven to provide highly stable indicators of latent attachment [56]. The classification of this questionnaire is based on the Bartholomew’s four-category model [57], which is obtained by the combined analysis of two dimensions: *Anxiety* (Cronbach’s α=0.93) and *Avoidance* (Cronbach’s α=0.85). Individuals with high levels of *Anxiety* are typically preoccupied with relationships, jealous, fearful of being abandoned or rejected (i.e., “I often worry that my partner does not really love me.”). High levels of *Avoidance* are evident in people who are fearful of intimacy, and tend to rely mainly on themselves more than on the partner (i.e., “I am nervous when partners get too close to me.”).

### 2.4. Genetics: rs25531

To assess Instagram users’ genotypes, the procedure described by Bonassi et al. [45] was also applied to the current study. ACGT, Inc. (Wheeling, IL, USA) conducted both the DNA extraction and its genotyping. The DNA extraction required the use of Oragene DNA purification reagent, whose concentrations were calibrated by using spectroscopy (NanoDrop Technologies, Wilmington, DE 19810, USA). Polymerase chain reaction (PCR) amplified each DNA sample for the *5-HTTPLR* gene rs25531 region target with the primers 5-GGCGTTGCCGCTCTGAATGCC-3 and 5-GAGGGACTGAGCTGGACAACCAC-3. A PCR reaction of 20 ll, comprising 1.5 ll of genomic DNA from the test sample, PCR buffer, 1 mM each of the forward and reverse primers, 10 mM deoxyribonucleotides, KapaTaq polymerase, and 50 mM MgCl2 was executed. PCR operation comprised 15 min denaturation at 95 °C, and 35 cycles at 94 °C (30 s), 60 °C (60 s), 72 °C (60 s) and a final 10 min step at 72 °C. PCR reactions were genotyped using an ABI 3730xl Genetic Analyzer (Applied Biosystems Inc., 2665 NN Bleiswijk, The Netherlands) and normalized with GeneScan 600 LIZ (Applied Biosystems, Inc., 2665 NN Bleiswijk, The Netherlands) size standards on each sample. GeneMapper ID (Applied Biosystems, Inc., 2665 NN Bleiswijk, The Netherlands) was employed to inspect Genotypic data.

Consistent with the Hardy–Weinberg Equilibrium ($X^{2}$ (1) = 1.309, ns), the distribution of participants’ alleles was as follows: T/T = 42, T/C = 15, C/C = 0. Participants having at least a C allele (C/C and T/C) were grouped into the C-carriers group. Thus, the final allelic frequency in this sample was 42 (73.68%) for T/T and 15 (26.32%) for C-carriers in line with the average distribution of the genotypes in the South-East Asiatic population. Moreover, participants’ age (*t*(55) = 0.915, ns) and gender ($X^{2}$(1) = 0.6343, ns) did not significantly differ between the two groups T/T vs. C-carriers.

### 2.5. Instagram Behavior

Three metrics from each Instagram user’s profile were assessed: posts, followings and followers. Firstly, the number of published posts represents an estimate of photos or videos uploaded by the user in their personal profile and shared with the Instagram community. Secondly, the number of followed profiles (called “followings”) indicates the number of accounts each user follows. Note that several and diverse reasons drive the choice of the accounts to follow. A given Instagram account may be managed by a person who feeds private life content or even by an industry that sponsors their product. Likewise, an Instagram account may be committed to specific contents (e.g., educational, humorous). Thirdly, the number of followers reveals the number of users that follow each participant’s account. In summary, a high number of posts is considered a measure of social hyperactivity. A high number of followings reveals a significant prosocial interest towards other users or their posts, and a high number of followers discloses a strong social ability to attract other users and influence the network. A fourth index, called a Social Desirability Index (SDI), was finally computed from the ratio between followers and followings. From previous works [45,52], SDI is calculated as a measure of general sociability and users’ network size. High values of SDI suggest a significant difference between the number of followers and the number of followings. Low values of SDI mean that the number of followers is approximately close to the number of followings, thus revealing a symmetric and balanced network between the social contacts to which the posts are addressed (followers) and the social contacts to which the user is inspired (followings). In summary, this index suggests how many followers are reached at the expense of the number of followings.

### 2.6. Statistical Analysis

R (R-core base version 4.0.0. Windows version) was used for statistical analysis and graphics. Instagram data were normalized by z-scores, and a preliminary analysis of outliers was conducted. Eight outliers were detected (posts: 2; followings: 3; SDI: 2; followers: 1) as distant at least 2 *SD* from the mean of each Instagram distribution. Following the winsorization approach [58,59], each outlier’s value was replaced by the mean of the observations for each dependent variable (here the Instagram variables) with outliers omitted. At this point, both the ECR-R and Instagram distributions were visualized with quantile–quantile and density plots and tested for normality, and their skewness and kurtosis were calculated (see Table 1).

Only Instagram number of posts did not present a Gaussian distribution. Therefore, log-transformation was applied to the variable number of posts to normalize the distribution of data (see Table 1).

## 3. Results

Three gene*environment interactions (rs25531 * close relationship in adulthood) on the number of Instagram posts, followings and SDI were hypothesized. Independent of gender, participants with the T/T genotype were expected to have fewer posts, followings and SDI values when they reported low scores in the ECR-R dimensions compared to C-carriers, whereas potential effects on Instagram number of followers were examined at an exploratory level.

One preliminary Student’s *t*-test was performed on each Instagram variable to probe if online users’ behaviors were affected by their gender (corrected α=0.0125). Preliminary results showed no significant differences between female and male users on the number of posts (*t*(55) = 0.669, *p* = 0.506), number of followings (*t*(55) = 2.513, *p* = 0.015), SDI (*t*(55) = 0.889, *p* = 0.377) and the number of followers (*t*(55) = 1.814, *p* = 0.075). As a result, gender was not included as a between-subjects factor in the subsequent ANCOVA models on the Instagram variables.

The same statistical approach with a differential Bonferroni correction was applied to those Instagram variables (number of posts, followings and SDI; corrected α=0.017) tested for the analysis driven by the hypothesis and the Instagram number of followers, which was considered only for exploratory analysis (α=0.05). One mixed ANCOVA was computed for each Instagram variable with the Instagram variable as the dependent variable, the genotype rs25531 (T/T vs. C-carriers) as a between-subject factor and the ECR-R dimensions (Anxiety and Avoidance) as continuous covariates. Bartlett’s tests confirmed the assumption of homogeneity of variance on the Instagram metrics. Partial eta squared and Cohen’s *d* were implemented to estimate the magnitude of the significant effects observed from the inferential tests (ANCOVA and Welch’s *t*-*test, respectively).*

A sensitivity power analysis for the ANCOVA using G*Power (version 3.1) given power equal to 0.80 and α error probability set at 0.017 (only hypothesis-driven analysis) for a sample of 57 participants previously estimated large effect size (f = 0.44).

In contrast with the hypothesis, no interactions between the genetic factor and the ECR-R dimensions were found on the three predicted Instagram variables number of posts, followings and SDI (see Table 2 and Tables S1 and S2 of the Supplementary Materials).

From all the three ANCOVAs, one main effect of ECR-R avoidance was detected on the Instagram number of followings (*F*(1,51) = 6.301, *p* = 0.015, p$\eta^{2}$ = 0.110) (see Table 2).

**Figure 1:**
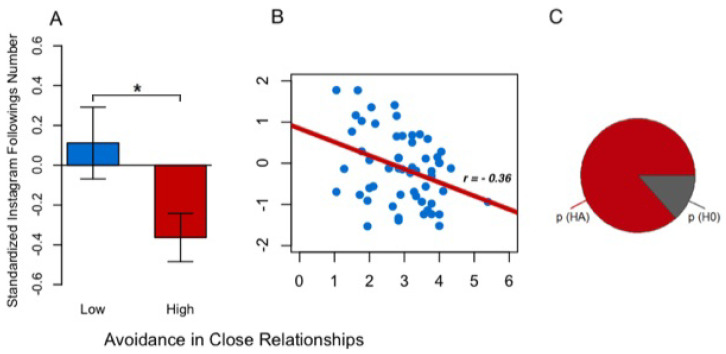
Effect of the avoidance on the standardized Instagram number of followings. (**A**) Contrast between low (blue) and high (red) scores pf ECR-R avoidance on Instagram number of followings. (**B**) Linear model with ECR-R Avoidance as continuous predictor of Instagram number of followings as continuous dependent variable. (**C**) Positive evidence for the alternative model HA (pBIC = 0.864; red area) and weak evidence for the null model H0 (pBIC = 0.135; grey area). (* *p* < 0.05).

The sample was successively separated into two groups (low vs. high ECR-R dimension) by the median split procedure. The distinct direction of the effect was confirmed by a Welch’s *t*-test, which revealed that users reporting low scores in avoidance displayed a greater number of followings than those who reported high scores in avoidance (*t*(48) = 2.190, *p* = 0.033; *d* = 0.576) (see Figure 1A).

The strength of the prediction of ECR-R avoidance on Instagram number of followings was validated post hoc (Figure 1B) with the support of a basic Bayesian approach as an alternative to the common inferential method [60,61]. The Bayesian information criterion (BIC) index (see the Formula (S1) at the Supplementary Materials for a definition of the BIC given L as the maximum likelihood function of the data, *k* as the number of free parameters and *n* as the number of observations) was computed for the alternative model HA (*k* = 3, *n* = 57) and the null model H0 (*k* = 2, *n* = 57) [62]. The differential BIC (ΔBIC) index between the two models was obtained, and a simple approximation of the Bayes Factor was calculated to derive the posterior BIC for the null model and the alternative model (see the Formulas (S2)–(S4) at the Supplementary Materials) [62,63,64]. Estimates of the posterior BIC were finally depicted on a pie chart to provide an immediate comprehension of the evidence in support of each model.

Employing the BIC we compared two statistical models of linear regression (BF = 0.156, ΔBIC = −3.710): the model of the alternative hypothesis (HA: Instagram number of followings is predicted by avoidance scores) vs. the model of the null hypothesis (H0: Instagram number of followings is predicted by the intercept only). We found positive evidence [65] for the model of the alternative hypothesis (BIC = 146.885, pBIC = 0.865) against the model of the null hypothesis (BIC = 150.565, pBIC = 0.135) (Figure 1C). Avoidance from the partner resulted to be a linear predictor of Instagram number of followings (Figure 1B). Pearson’s *r* correlation between the same ECR-R dimension and the Instagram variable was also determined. As is evident from Table 3, avoidance in close relationship was negatively associated with the Instagram number of followings (*t*(55) = −2.831, *r* = −0.36, *p* = 0.007) (Figure 1B; Table 3).

No other significant main effects emerged for number of posts and SDI, respectively, (see Table 2 and Tables S1 and S2 of the Supplementary Materials).

At an exploratory level, one further mixed ANCOVA was calculated on the Instagram number of followers (α=0.05). No significant main effects or interaction effects between genotype and ECR-R dimensions were identified on the Instagram number of followers (see Table S3 of the Supplementary Materials).

Potential associations between Instagram variables and ECR-R dimensions were finally scrutinized by exploratory Pearson’s correlation tests. Table 3 reports the exploratory intercorrelations among all the continuous variables. As regards the intracorrelations among Instagram variables, the number of posts was positively associated with the number of followings (*t*(55) = 2.596, *r* = 0.33, *p* = 0.012) and followers (*t*(55) = 2.354, *r* = 0.30, *p* = 0.022). The number of followers was also significantly and positively correlated with the number of followings (*t*(55) = 10.670, *r* = 0.82, *p* = 0.000) and the SDI (*t*(55) = 3.052, *r* = 0.38, *p* = 0.003). As regards the intracorrelations among ECR-R dimensions, a positive relationship between anxiety and avoidance was ascertained (*t*(55) = 3.652, *r* = 0.44, *p* = 0.000). Regarding the intercorrelations among Instagram variables and ECR-R dimensions, only the negative association between the number of followings and avoidance was significant, as reported in the previous subsection.

## 4. Discussion

The first goal of the current research was to inspect at what extent the genetic predisposition of the serotonin transporter gene and the felt quality in close relationships could influence the online sociability on a SNS like Instagram. Given the rs25531 as a genetic component and the perceived adult attachment with the romantic partner as a proxy of the social environment, one genotype × environment interaction was predicted on Instagram number of posts, followings and SDI independent of gender. One separate and exploratory genotype × environment interaction was also tested on Instagram number of followers.

As predicted, male and female Instagram users did not show differences in the four Instagram metrics. In contrast to the hypothesis, no interaction between rs25531 polymorphisms and patterns in close relationships was identified for Instagram number of posts, followings, SDI or followers. However, participants’ score on the ECR-R dimension avoidance was found to modulate the Instagram number of followings. At a statistical view, only this effect is deemed significant using the Bonferroni’s corrected alpha threshold. Subsequent tests confirmed that adult avoidance was negatively associated with the number of followings: the higher the reported levels of adult avoidance, the lower the number of followed people on Instagram. Specifically, Instagram users who experienced a lower avoidance towards the partner displayed a greater quantity of Instagram followings than those who felt higher avoidance. Post-hoc verification by a Bayesian method finally demonstrated the positive and prevalent evidence of the linear relationship between avoidance and Instagram number of followings when contrasted with the controlled weak evidence of null relationship between the same continuous variables.

Given the multifactorial nature of the research, various considerations can be advanced depending on the lens through which data are observed.

Firstly, the developmental psychology approach drives the attention to directional effect of adult attachment on Instagram behavior. Generally, people involved in high quality marriages show more positive outcomes when compared to the protagonists of unsatisfying unions [66,67]. Specifically, partnership quality, besides moderating the effects of a romantic relationship on psychological well-being [68,69,70,71], has a role on influencing the person’s social life. For instance, the reasons why individuals refrain from partnerships differ according to their life experiences as well as their personality traits (e.g., withdrawal, suspiciousness, insensibility), feelings or even clinical conditions (e.g., depressive or anxiety disorders) [72,73,74,75]. In the context of offline social interactions, marital discord can lead to a greater social role impairment in relationships with relatives and friends [76,77]. Concerning the online social interactions probed in this study, users with high levels of avoidance perceived in the relationship with the partner were inclined to minimize their number of followed people on Instagram. This finding corroborates with the comparable pattern described by Lee [78], who found a negative correlation between avoidant attachment and the propensity to social bonding on a SNS. Accordingly to previous studies, anxiety in close relationships positively predicts SNSs addiction [79], whereas avoidance in close relationships is negatively associated with SNSs addiction [80]. Users who tend to evade close relationships are not more interested by alternative social activities either in offline or online life [81,82,83]. In reference to Instagram, the distress resulting from the fear of intimacy in the couple could be persistent in in-person social circumstances with friends but also virtual social places with followings. Therefore, users’ showing an avoidant pattern from other “Instagrammers” probably echoes the avoidant attachment with their romantic partner. This being said, the general propensity to avoid others in social interactions could be explained by negative representation of conspecifics and unfavorable internal working models that prevent the individuals from trust and social engagement [78,84,85]. It is likely to assume that avoidant users are inclined to social withdrawal from collective situations that could evoke a sense of discomfort and inappropriateness [86]. As a consequence, avoidant users could probably spend short periods in proximity with their partner and a small amount of time is dedicated to online social activities [87].

At the same time, Instagram users who revealed a more secure attachment in terms of low scores in avoidance could be predisposed to social commitment in offline and online social environments. Furthermore, the sense of fit and affection felt by these users when they are close to their partner could lead them to a greater Instagram usage. The supportive and comforting conduct with the partner could persuade non-avoidant Instagram users to search for others, increasing the number of followings, and visualize their posts (i.e., published content as videos and pictures). As prosocial agents of the SNS, such users could be interested in developing new virtual social ties [88].

Overall, the findings seem to extend the influence of the quality of close relationships even to SNS, such as Instagram [89,90,91,92]. The same findings agree with previous evidences, which highlighted an increased propensity of anxious users to search for approval of other contacts, but a decreased approaching behavior of avoidant users to others [93,94].

A more complex explanation could be offered when the findings are examined through the lens of behavioral genetics. Here, Instagram users’ score on the ECR-R dimension avoidance towards the partner predicted the Instagram number of followings independently of the genetic profile. Based on these outcome, Instagram social behavior could be predominantly affected by the environmental exposure throughout human development with no differences in the biological inclination to online sociability among genetic carriers of the serotonin transporter gene. Within the genotype-by-environment framework, no influence of the specific genetic predisposition from the rs25531 was ascertained, but adult avoidance from the partner considerably affected the change in the Instagram number of followings. The effect of the environmental action on the offline social behavior across the lifespan independent of the genetic influences is effectively documented in literature [95,96,97]. However, only a minority of studies investigated the peculiar role of rs25531 in offline as well as online behavior. For instance, a previous study [45] found that the interaction between rs25531 and early attachment with parents modulated the Instagram SDI. On the basis of this evidence, it is worth noting that rs25531 genetic predisposition could moderate the social sensitivity in offline and online environments only when combined with experiential factors in childhood (e.g., parental bonding) [38,45,98,99] and adulthood as regards the peer relationships [46], but not necessarily in close relationships, as pointed by the present work.

In light of the considerations discussed, the current research provides new evidence on the interplay between genetic factors within rs25531 and adult attachment with the partner on Instagram online sociability.

## 5. Limitations, Conclusions and Future Directions

The present study has some limitations that require a careful evaluation. Firstly, according to the statistical requirements applied to candidate gene association studies, the sample of participants involved in this study was limited and largely composed of females. Although a rigorous statistical procedure was adopted to decrease the likelihood of committing Type I error, a small sample size could increase the probability of a Type II error and reduce the power, thus pointing to less conclusive results.

Overall, the present findings should be treated with caution. However, the sensitivity analysis for the ANCOVA showed evidence that the effect size was consistent with the power estimation. Furthermore, the application of a Bayesian approach as an alternative to the classical inferential statistics ensured a high and bidirectional control of the evidence in favor of the alternative hypothesis rather than the null hypothesis.

Secondly, a convenience sample of young university students was used. Thirdly, the adult attachment in close relationships was assessed by a self-report measure that could be sensitive to the subjective feelings or judgments of the participant. Thirdly, the genetic factors of the region rs25531 are here considered with no reference to the short/long allelic variation of the serotonin transporter gene. Moreover, participants’ Instagram number of followings and followers were collected but not controlled for their role in the social network. For instance, among the number of followings could be included not only private users, but also company profiles of e-shops or brands. The same content could vary across Instagram profiles, from daily moments to humorous content or products’ sponsorship. Lastly, participants’ personality traits as potential factors of influence on the attachment styles and Instagram activity were not assessed.

The association of 5-HTTLPR/rs25531 and psychological and behavioral traits is still quite contentious in the literature [100]. Future studies should consider a larger sample size to try to balance the number of participants between groups (i.e., gender, age, ethnicity, education) and conditions (genetic groups and attachment levels). Starting from the linkage (1) between serotonin reuptake and major depression [39] and, simultaneously, (2) between Instagram usage and depressive symptoms [101], a future model could control how the interaction between genetic factors of 5-HTTLPR/rs25531, adult attachment and vulnerability to depression could impact on the Instagram responses. At this level, the interaction between genotype and environment could be intended as a statistical interaction, as explored in this study, and could imply a systemic interaction at a broader biological level. Individuals from different countries could be involved in a cross-cultural investigation to test potential culture-by-genotype interactions on social media behavior. Alternative SNPs could also be analyzed in relation with online social behavior. Although the increasing amount of literature on Facebook in the last decade, a direct gene-by-environment investigation is still missing for Facebook social activity. A cross-comparison among personality traits and social metrics (e.g., friends or followings and followers) of different SNSs, such as Facebook, Instagram, LinkedIn, Snapchat, TikTok could be advanced under a multifactorial perspective. All these suggestions could address the next multidisciplinary researches into the mechanisms underlying online socialization on digital platforms.

## Figures and Tables

**Table 1:** Summary of the descriptive statistics for each continuous variable. The distributions of ECR-R (Anxiety and Avoidance) and Instagram metrics (number of posts, followings, Social Desirability Index and followers) are described in terms of Minimum (Min), first Quartile (1st Q), Median, Mean, third Quartile (3rd Q), Maximum (Max), Skewness, Kurtosis and Standard Deviation (SD). Log-transformed number of posts shows increased values compared to the original sampling of the same variable.

Variable	Min	1st Q	Median	Mean	3rd Q	Max	Skewness	Kurtosis	SD
Anxiety	1.39	3.33	3.94	3.94	4.56	6.22	−0.38	−0.02	1.08
Avoidance	1.06	2.11	2.94	2.95	3.67	5.39	−0.15	−0.39	0.93
Posts number	−0.60	−0.51	−0.36	−0.16	−0.02	1.36	1.56	1.81	0.49
Log−transformed posts number	0.33	0.40	0.49	0.58	0.68	1.21	1.14	0.43	0.23
Followings	−1.53	−0.77	−0.12	−0.12	0.51	1.78	0.35	−0.62	0.85
SDI	−1.16	−0.27	−0.13	−0.16	−0.03	0.61	−0.70	2.65	0.29
Followers	−0.80	−0.50	−0.17	−0.11	0.22	1.45	0.78	0.16	0.51

**Table 2:** Results and effect sizes of hypothesis-driven ANCOVA computed for Instagram number of followings. Degrees of freedom (DF), sum square, mean square, *F* value, *p*-value and partial eta squared (p$\eta^{2}$) are reported for main and interaction predictors. A significant main effects of avoidance was found on the number of followings. (* *p* < 0.05).

Variable	DF	Sum Square	Mean Square	*F* Value	*p*	p$\eta^{2}$
*5-HTTLPR*/rs25531	1	0.460	0.456	0.684	0.412	0.013
Anxiety	1	1.520	1.517	2.278	0.137	0.043
Avoidance	1	4.20	4.196	6.301	0.015 *	0.110
*5-HTTLPR*/rs25531 x Anxiety	1	0.480	0.481	0.723	0.399	0.014
*5-HTTLPR*/rs25531 x Avoidance	1	0.030	0.028	0.041	0.840	0.001
Residuals	51	33.960	0.666			

**Table 3:** Pearson’s *r* values and significance levels among Instagram number of posts (Posts_N), number of followings (Followings_N), SDI, number of followers (Followers_N) and ECR-R Anxiety and Avoidance. (* *p* < 0.05, ** *p* < 0.01, **** *p* < 0.0001).

*Variable*	Posts_N	Followings_N	SDI	Followers_N	Anxiety	Avoidance
**Posts_N**		0.33 *	0.02	0.30 *	0.05	−0.19
**Followings_N**			0.21	0.82 ****	−0.21	−0.36 **
**SDI**				0.38 **	−0.18	−0.12
**Followers_N**					−0.19	−0.26
**Anxiety**						0.44 ****
**Avoidance**						

## Data Availability

The dataset generated for this study can be found in the in the NTU’s Data repository (DR-NTU Data) at the following address: https://doi.org/10.21979/N9/FRFMXV.

## Supplementary Materials

The following are available at https://www.mdpi.com/article/10.3390/ijerph18147547/s1, Formula (S1): Definition of the considered Bayesian Information Criterion. Formula (S2): Derivation of the Bayesian Factor from the Bayesian Information Criterion. Formula (S3): Posterior probability of the null model. Formula (S4): Posterior probability of the alternative model. Table S1: ANCOVA on Instagram number of posts. Table S2: ANCOVA on Instagram SDI. Table S3: ANCOVA on Instagram number of followers.

## Abbreviations

SNS	Social Networking Site
*5-HTTLPR*	*Serotonin Transporter Gene*
SNP	Single Nucleotide Polymorphism
ECR-R	Experience in Close Relationship-Revised
SDI	Social Desirability Index
