# Supplementary material for: Modulation of Instagram Number of Followings by Avoidance in Close Relationships in Young Adults under a Gene x Environment Perspective
Source: Int J Environ Res Public Health. 2021 Jul 15;18(14):7547. doi: 10.3390/ijerph18147547 (PMC8303232; doi:10.3390/ijerph18147547)
Supplement: Supplementary file 1 [file ijerph-18-07547-s001.zip › ijerph-1258910-supplementary.pdf]

Supplementary Formulas

Formula S1: Definition of the considered Bayesian Information Criterion

$$BIC = -2 * ln(L) + k * ln(n) \tag{1}$$

Formula S2: Derivation of the Bayesian Factor from the Bayesian Information Criterion. Note: this formula represents a basic approximation which was sufficient for the theoretical hypothesis of this research

$$BF = e^{[BIC(HA) - BIC(HO]/2} \tag{2}$$

Formula S3: Posterior probability of the null model

$$pBIC(H0) = \frac{BF}{(BF + 1)} \tag{3}$$

Formula S4: Posterior probability of the alternative model

$$pBIC(HA) = 1 - pBIC(H0) \tag{4}$$

Supplementary Tables

Table S1: ANCOVA on Instagram number of posts

Table 1: Results and effect sizes of hypothesis-driven ANCOVA computed for Instagram number of posts. Degrees of freedom (DF), sum square, mean square, F value, p-value and partial eta squared ($p\eta^2$) are reported for main and interaction predictors.

**Variable**	**DF**	**Sum Square**	**Mean Square**	F **value**	p**-value**	$p\eta^2$
5-HTTLPR/rs25531	1	0.014	0.014	0.261	0.612	0.005
Anxiety	1	0.006	0.006	0.111	0.741	0.002
Avoidance	1	0.154	0.154	2.822	0.099	0.052
5-HTTLPR/rs25531 x Anxiety	1	0.028	0.028	0.508	0.479	0.010
5-HTTLPR/rs25531 x Avoidance	1	0.044	0.044	0.803	0.374	0.016
Residuals	51	2.787	0.055			

Table S2: ANCOVA on Instagram SDI

Table 2: Results and effect sizes of hypothesis-driven ANCOVA computed for Instagram SDI. Degrees of freedom (DF), sum square, mean square, F value, p-value and partial eta squared ($p\eta^2$) are reported for main and interaction predictors.

**Variable**	**DF**	**Sum Square**	**Mean Square**	F **value**	p**-value**	$p\eta^2$
5-HTTLPR/rs25531	1	0.107	0.107	1.213	0.276	0.023
Anxiety	1	0.126	0.126	1.428	0.238	0.027
Avoidance	1	0.027	0.027	0.308	0.581	0.006
5-HTTLPR/rs25531 x Anxiety	1	0.012	0.012	0.134	0.716	0.003
5-HTTLPR/rs25531 x Avoidance	1	0.001	0.001	0.007	0.934	0.000
Residuals	51	4.513	0.089			

Table S3: ANCOVA on Instagram number of followers

Table 3: Results and effect sizes of exploratory ANCOVA computed for Instagram number of followers. Degrees of freedom (DF), sum square, mean square, F value, p-value and partial eta squared ($p\eta^2$) are reported for main and interaction predictors.

**Variable**	**DF**	**Sum Square**	**Mean Square**	F **value**	p**-value**	$p\eta^2$
5-HTTLPR/rs25531	1	0.157	0.157	0.618	0.436	0.012
Anxiety	1	0.470	0.470	1.852	0.180	0.035
Avoidance	1	0.660	0.660	2.600	0.113	0.049
5-HTTLPR/rs25531 x Anxiety	1	0.028	0.028	0.109	0.743	0.002
5-HTTLPR/rs25531 x Avoidance	1	0.041	0.041	0.161	0.690	0.003
Residuals	51	12.937	0.254			
